# Activating *MAPK1* (ERK2) mutation in an aggressive case of disseminated juvenile xanthogranuloma

**DOI:** 10.18632/oncotarget.17521

**Published:** 2017-04-29

**Authors:** Rikhia Chakraborty, Oliver A. Hampton, Harshal Abhyankar, Daniel J. Zinn, Amanda Grimes, Brooks Skull, Olive Eckstein, Nadia Mahmood, David A. Wheeler, Dolores Lopez-Terrada, Tricia L. Peters, John M. Hicks, Tarek Elghetany, Robert Krance, Poulikos I. Poulikakos, Miriam Merad, Kenneth L. McClain, Carl E. Allen, Donald W. Parsons

**Affiliations:** ^1^ Texas Children's Cancer Center, Texas Children's Hospital, Houston, TX 77030, USA; ^2^ Department of Pediatrics, Division of Pediatric Hematology-Oncology, Baylor College of Medicine, Houston, TX 77030, USA; ^3^ Department of Molecular and Human Genetics, Baylor College of Medicine, Houston, TX 77030, USA; ^4^ Department of Pathology and Immunology, Baylor College of Medicine, Houston, TX 77030, USA; ^5^ Human Genome Sequencing Center, Baylor College of Medicine, Houston, TX 77030, USA; ^6^ Body and Nuclear Radiology Sections, Texas Children's Hospital, Houston, TX 77030, USA; ^7^ Center for Cell and Gene Therapy, Houston, TX 77030, USA; ^8^ Department of Oncological Sciences, Icahn School of Medicine, New York, NY 10029, USA; ^9^ Tisch Cancer Institute, Icahn School of Medicine, New York, NY 10029, USA; ^10^ Immunology Institute, Icahn School of Medicine, New York, NY 10029, USA; ^11^ Department of Dermatology, Icahn School of Medicine, New York, NY 10029, USA

**Keywords:** juvenile xanthogranuloma, *MAPK1*, ERK activation, histiocytic disorder, somatic mutation

## Abstract

Juvenile xanthogranuloma (JXG) is a rare histiocytic disorder that is usually benign and self-limiting. We present a case of atypical, aggressive JXG harboring a novel mitogen-activated protein kinase (MAPK) pathway mutation in the *MAPK1* gene, which encodes mitogen-activated protein kinase 1 or extracellular signal-regulated 2 (ERK2). Our analysis revealed that the mutation results in constitutive ERK activation that is resistant to BRAF or MEK inhibitors but susceptible to an ERK inhibitor. These data highlight the importance of identifying specific MAPK pathway alterations as part of the diagnostic workup for patients with histiocytic disorders rather than initiating empiric treatment with MEK inhibitors.

## INTRODUCTION

Juvenile xanthogranuloma (JXG) is a rare non-Langerhans cell (LCH) histiocytic disorder with histologic features characteristic of dermal dendrocytes (fascin^+^, CD1a^−^, CD207^−^) and macrophages (CD14^+^, CD68^+^, CD163^+^, and factor XIIIa+) [1]. While the majority of patients with JXG have skin-limited lesions, some also present with disseminated disease [2]. JXG is histologically similar to Erdheim-Chester disease (ECD), a disseminated histiocytic disorder of adults that is also characterized by frequent activating somatic mutations of MAPK pathway genes [3, 4]. Pediatric JXG has also been reported in children with germline mutations in the neurofibromatosis 1 (*NF1*) or neurofibromatosis 2 (*NF2*) genes [5, 6]. We have previously described two patients with multifocal histiocytic lesions with characteristics of both LCH and JXG that harbor the *BRAF-V600E* mutation [7]. Mixed histiocytic disorders with phenotypic features of more than one classic histology (e.g. CD207+/fascin- and CD207-/fascin+ in a single lesion) or lesions with multiple distinct histologies (e.g. homogenous CD207+ or fascin+ in different lesions) with common mutations across lesions are an increasingly recognized phenomenon [8, 9]. These data support a model of pathogenesis in which activating MAPK pathway mutations occurring at specific stages of differentiation in myeloid precursor cells define the histologic and clinical presentations of diverse histiocytic disorders [8]. Here we present a case of a patient with atypical, aggressive JXG harboring a novel mitogen-activated protein kinase (MAPK) pathway mutation in the *MAPK1* gene, which encodes mitogen-activated protein kinase 1 or extracellular signal-regulated 2 (ERK2).

## RESULTS

In this case, a 10 year old previously-healthy male patient presented with shortness of breath and a large mediastinal mass. Multiple enlarged lymph nodes (mediastinal, hilar, abdominal, and pelvic) and splenomegaly were noted on CT scan. Further staging identified infiltrative lesions involving the liver, spleen, bone marrow and lungs. Histologic examination of a pre-therapy lymph node biopsy specimen revealed non-caseating granulomas with sheets of CD1A^−^/CD207^−^/fascin^+^/CD163^+^/factorXIII^−^/PGM1^+^ histiocytes (Supplementary Figure 1A). Based on overall histology and immunohistochemistry, this case was thought to most closely resemble JXG. Cytogenetic and fluorescence *in situ* hybridization (FISH) evaluation of the lymph node biopsy specimen identified a clonal population with complex karyotype including three copies of the *IGH* gene in 95/200 (47.5%) interphase cells examined (Supplemental Data; Supplementary Table 1).

Treatment with clofarabine initially decreased the lymphadenopathy and splenomegaly. After two months of treatment the patient relapsed with fever, lymphadenopathy, hepatosplenomegaly and prolonged pancytopenia, and became transfusion dependent for both red blood cells and platelets. The bone marrow showed patchy histiocytic proliferation with formation of non-caseating granulomas, identical to the original bone marrow studies. Over the course of 7 months the patient had incomplete responses to the following chemotherapy regimens: methotrexate, etoposide, ifosfamide and dexamethasone; alemtuzumab; bortezomib, vinorelbine and ifosfamide. After failing these treatments he received etoposide and dexamethasone for 5 months achieving nearly a complete remission, then received myeloablative conditioning and stem cell transplant resulting in complete remission (Supplementary Figure 1B). He remained in remission but died post-transplant from acute respiratory failure of uncertain etiology, most likely infection complicated by acute pulmonary hemorrhage approximately 3 months after transplant.

Blood and frozen tumor samples were collected at the time of diagnosis (before initiation of treatment) and post-chemotherapy under a Baylor College of Medicine IRB-approved protocol and DNA was extracted. Whole exome sequencing (WES) on blood and tumor was performed as previously described [7] using the Baylor College of Medicine Human Genome Sequencing Center VCRome 2.1 design array (42 Mb, NimbleGen) on an Illumina HiSeq 2000 instrument platform and analyzed utilizing the HGSC Mercury pipeline (https://www.hgsc.bcm.edu/software/mercury) with 96.27% of target bases having at least 20-fold coverage.

**Figure 1:**
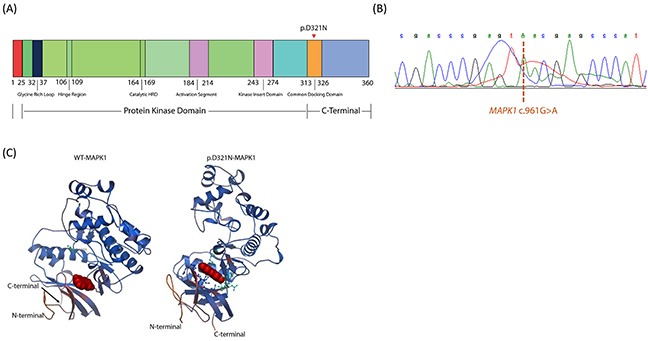
**(A)** Schematic illustration of the functional domains in human ERK2. Triangle indicates the missense mutation identified in the current study. Figure not to scale. **(B)** Electrophoretogram obtained from Sanger sequencing of the *MAPK1* gene in the patient. The dashed line indicates the c.961G>A point mutation detected. **(C)** Ribbon diagrams of wild-type and mutated human ERK2 proteins depicting predicted structural changes resulting from the identified *MAPK1* mutation, including alterations in the C-terminal docking (CD) domain. Structures were prepared from protein data bank file 4S31.

A novel somatic mutation was detected in *MAPK1*, the gene which encodes p42MAPK or ERK2. The mutation (c.961G>A; p.D321N) localizes to the C-terminal docking (CD) domain of ERK2 (Figure 1A) and was observed in 7/71 (10%) reads from the tumor and 0/102 from the matched peripheral blood sample. It was confirmed by sequencing on the Ion AmpliSeq platform (182/1996 variant reads in tumor versus 9/1429 variant reads in normal) [7] as well as targeted PCR and Sanger sequencing (Figure 1B). Three other somatic mutations in genes not known to be linked to cancer were also identified (Supplementary Table 2).

**Figure 2:**
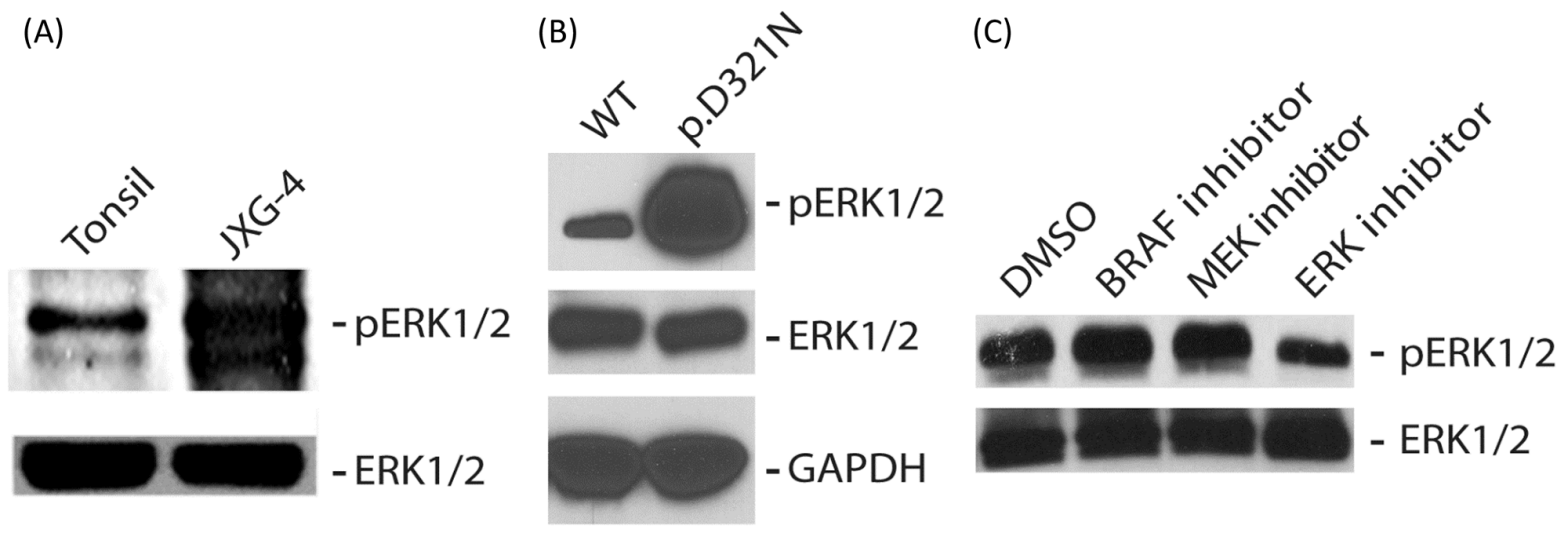
**(A, B)** Immunoblot analysis of P-ERK and total ERK in whole cell lysate obtained from patient lesion **(A)** or HEK293 cells transiently transfected with plasmids expressing either wild type or p.D321N ERK2 protein **(B)**. GAPDH served as a loading control in ‘**B**’. **(C)** Immunoblot analysis of P-ERK and total ERK in whole cell lysate obtained from the patient lesion treated for 4 hours with either 200 nM of the *BRAF*-V600E inhibitor vemurafenib, or 10 nM of the MEK inhibitor trametinib (Selleckchem, Houston, TX), or 40 nM of the ERK inhibitor TCS ERK 11e (Tocris Bioscience, Bristol, UK).

*In silico* analysis of the predicted 3-dimensional structures of wild-type and p.D321N mutant proteins using SWISS-MODEL and Swiss-PdbViewer [10] revealed changes in the CD domain of ERK2 (Figure 1C). To assess the functional effect of the p.D321N mutation on the MAPK pathway, we analyzed the phosphorylation status of ERK1 and ERK2 in primary cell culture from the patient's tumor biopsy compared to healthy control tonsil (from elective tonsillectomy), and also in HEK293 cells transiently transfected with wild-type or p.D321N mutant *MAPK1* constructs generated by site-directed mutagenesis. In both cases, the mutation led to constitutive ERK activation, in contrast to either the healthy tonsil tissue specimen or the transfected wild-type ERK2 construct. (Figure 2A, 2B).

We next assessed the ability of various targeted MAPK inhibitors to suppress pathway activation induced by the *MAPK1* mutation. As would be predicted from canonical MAPK signalling, ERK phosphorylation was unaffected by a *BRAF*-V600E inhibitor (vemurafenib) or a MEK inhibitor (trametinib) [11, 12]. The ERK inhibitor (TCS ERK 11e) was able to decrease ERK1/2 phosphorylation (Figure 2C).

## DISCUSSION

Our previous WES analysis of 4 JXG cases with classic morphology (2 skin-limited and 2 disseminated) and 3 mixed LCH/JXG lesions did not reveal any somatic mutations in MAPK genes other than *BRAF*-V600E in mixed JXG/LCH lesions. However, a total of 17 somatic mutations in non-MAPK genes were found in the 4 “pure” JXG lesions analyzed (median of 4 mutations per case, range 0 to 9 mutations), including a *PI3KCD* mutation in one patient [7]. In another study, combined whole exome sequencing in skin lesions from 1 pediatric JXG patient and transcriptome sequencing in 11 JXG patients (including 4 pediatric patients) identified somatic mutations in *MAP2K1* (27% of the cases), *KRAS* (18%), and *NRAS* (18%) [3]. PI3K pathway mutations have also been frequently observed in ECD [3, 9].

The c.961G>A *MAPK1* mutation has been previously detected in head and neck squamous cell carcinoma [13], oral squamous cell carcinoma [14], and ovarian mixed germ cell tumors [15]. According to the COSMIC database (http://cancer.sanger.ac.uk/cosmic/gene/analysis?ln=MAPK1, accessed on August 16, 2016), somatic mutations (>74% missense) in *MAPK1* have been reported in 89 unique samples, most frequently squamous cell carcinoma and more rarely in melanoma and lymphoid neoplasms, but none in JXG or any other histiocytic disorder.

The c.961G>A *MAPK1* mutation resulted in constitutive ERK2 activation. Constitutive activation of ERK2 might occur due to uncoupling and loss of sensitivity to inhibition by dual specificity phosphatases (DUSPs) [16, 17], or loss of binding to p90 ribosomal S6 kinases (RSK) that can inhibit nuclear translocation and function of ERK2 [18, 19] – each function being reliant on an intact CD domain of ERK2, which was disrupted by this particular mutation. MAPK pathway activation is known to play a critical role in regulating macrophage production in colony stimulating factor 1-dependent proliferation of bone marrow progenitors [20]. ERK hyperactivation thus can also result in aberrant development of the macrophage progenitors.

Inhibition of BRAF and MEK within the MAPK pathway did not inhibit ERK activation, which was in contrast partially inhibited by a direct ERK inhibitor. However, it is of note that decrease in ERK phosphorylation does not necessarily confirm decreased ERK catalytic activity as it can also reflect altered inhibitor-induced binding of ERK with upstream MEK [21]. It has been shown in the context of glioblastoma that cross-inhibitory regulation between the MAPK and PI3K/mTOR pathways by p70S6K contributes to both tumorigenic and self-renewal capacity of cancer stem-like cells [22]. In fact, the most robust results for glioblastoma were obtained when a combination of MAPK and PI3K/mTOR inhibitors was used in comparison to individual inhibitors [22]. Both MAPK and PI3K/mTOR signaling pathways have multiple nodes that are essential for feedback regulation and crosstalk with other signaling pathways. In fact, MAPK can regulate mTORC1 activation by TSC2 phosphorylation [23, 24]. Hence, it will be important to define signaling crosstalk in histiocytic disorders in order to better inform rational therapeutic design.

Activating mutations in MAPK pathway genes have been identified in most cases of LCH [2, 25]. Emerging data from a handful of JXG cases sequenced to date supports the pathologic activation of ERK as a driver of pathogenesis in this histiocytic disorder as well. While JXG is often self-limiting, there are also cases of fatal progression. To our knowledge, this is the first case of an activating mutation in the gene encoding ERK2, the distal member of the canonical MAPK pathway, in a histiocytic disorder. Targeted inhibition of MAPK signaling is a potentially promising strategy for patients with chemotherapy-refractory disease. Based on existing data in which all identified activating MAPK pathway mutations have been proximal to ERK, one could consider empiric treatment with a MEK inhibitor for such patients [3]. However, this case provides an example in which MEK inhibition would presumably be ineffective, supporting a strategy of identifying the specific MAPK pathway alterations in each case as part of the diagnostic workup for patients with histiocytic disorders.

## MATERIALS AND METHODS

### Sample acquisition

Blood and frozen tumor samples were collected from the patient at the time of diagnosis (before initiation of treatment) and post-chemotherapy under a Baylor College of Medicine IRB-approved protocol and DNA was extracted.

### Immunohistochemistry

FFPE tissue sections (10 μm) were de-paraffinized and rehydrated through a graded alcohol series. Antigen retrieval was performed on FFPE slides using 1X Target Retrieval Solution (Dako, Carpinteria, CA). Endogenous peroxidase was blocked using 3% hydrogen peroxide for 10 minutes. The slides were incubated overnight with primary antibodies against Fascin (PA0420, Leica Biosystems, Buffalo Grove, IL, as it is), CD207 (NCL-LANGERIN-U, Leica Biosystems, Buffalo Grove, IL, 1:100 dilution), CD1A (PA0553, Leica Biosystems, Buffalo Grove, IL, as it is), Factor XIII (PA0449, Leica Biosystems, Buffalo Grove, IL, as it is), CD163 (NCL-CD163, Leica Biosystems, Buffalo Grove, IL 1:1500 dilution), and CD-68PGM1 (M0876, Agilent Technologies, Santa Clara, CA 1:300 dilution) at 4°C. Following incubation with secondary antibodies at room temperature for 1 hour, immunoreactivity were detected using diaminobenzidine (Innovex Biosciences, Richmond, CA) as a chromogen. Slides were counter-stained with hematoxylin before imaged with Olympus BX51 microscope fitted with a DP26 camera.

### Whole exome sequencing

Whole exome sequencing (WES) on DNA isolated from blood and tumor was performed using the Baylor College of Medicine Human Genome Sequencing Center VCRome 2.1 design array (42 Mb, NimbleGen) on an Illumina HiSeq 2000 instrument platform and analyzed utilizing the HGSC Mercury pipeline (https://www.hgsc.bcm.edu/software/mercury) as previously described [7]. Orthogonal validation of putative mutations on the AmpliSeq mutation panel platform was also performed as previously described [7]. Primary and somatic mutation data analyses were done as previously described [7].

### *MAPK1* mutation validation sequencing

Semi-quantitative PCR was performed on cDNA from patient sample using One*Taq* DNA polymerase (New England Biolabs, Cambridge, MA) using the following primers (Sigma Aldrich, St. Louis, MO): Forward primer - 5′-gctcagttgttttgtgggtaagt-3′; Reverse primer - 5′-atggttggtccactgctggctga-3′. PCR amplicon products were purified using QIAquick PCR Purification Kit (Qiagen, Valencia, CA) and confirmed visually by resolving on an one percent agarose gel. Using our designed primer sequences, the amplicons underwent Sanger sequencing at the Lone Star Labs (Houston, TX).

### Structural prediction of mutant ERK2 proteins

The three dimensional (3D) protein structures of the wild-type and mutant ERK2 proteins were modeled and compared using SWISS-MODEL and Swiss-PdbViewer [10]. The crystal structure of the wild-type ERK2 (4S31) was used to pattern the mutant 3D structure.

### Expression constructs

The specific c.961G>A mutation in *MAPK1* was generated by QuickChange XL Site-Directed Mutagenesis Kit (Agilent Technologies, La Jolla, CA) in the full length *MAPK1* (#HG10030-M-F) vector (Sino Biological, Beijing, P.R. China) using the 5′-gagcagtattacgacccgagtaacgagcccatc-3′ sense and 5′-gatgggctcgttactcgggtcgtaatactgctc-3′ anti-sense primers and sequence verified.

### Cell culture, transfection and treatment

HEK293 cells (ATCC, Manassas, VA) were cultured in DMEM (Lonza, Walkersville, MD) supplemented with 10% FBS (Lonza), and 2 mM L-glutamine (Life Technologies, Carlsbad, CA). 1.5 × 10^6^ HEK293 cells were transfected using Lipofectamine 3000 (Life Technologies, Carlsbad, CA) following manufacturer's recommendations. Where indicated, the cells were treated for 4 hours with either 200 nM of the *BRAF*-V600E inhibitor vemurafenib, or 10 nM of the MEK inhibitor trametinib (Selleckchem, Houston, TX), or 40 nM of the ERK inhibitor TCS ERK 11e (Tocris Bioscience, Bristol, UK). Cells were harvested 48 hours post-transfection.

### Immunoblot analysis

Harvested HEK293 cells, JXG patient lesion biopsy cell suspension, and healthy control tonsil (from elective tonsillectomy) were lysed in M-PER Mammalian Protein Extraction Reagent (Thermo Scientific, Waltham, MA) containing Halt protease and phosphatase inhibitor cocktail (Thermo Scientific). Whole cell lysates were resolved on a Criterion TGX 10% gel (Bio-Rad), transferred to an Immobilon PVDF membrane (Millipore, Billerica, MA), and probed with antibodies recognizing ERK1/2 and phosphorylated ERK1/2 (Cell Signaling Technologies, Danvers, MA). Where indicated, blots was stripped and re-probed with antibody recognizing GAPDH (Cell Signaling Technologies) to serve as a loading control. HRP-linked secondary antibodies against mouse and rabbit IgG (Cell Signaling Technologies) were used after probing with primary antibody. The blots were imaged using Clarity Western ECL Substrate (Bio-Rad) and Blue Devil autoradiography film (Genesse Scientific, San Diego, CA).

## SUPPLEMENTARY FIGURE AND TABLES




