# Supplementary material for: Activating MAPK1 (ERK2) mutation in an aggressive case of disseminated juvenile xanthogranuloma
Source: Oncotarget. 2017 Apr 29;8(28):46065–70. doi: 10.18632/oncotarget.17521 (PMC5542249; doi:10.18632/oncotarget.17521)
Supplement: Supplementary file 1 [file oncotarget-08-46065-s001.pdf]

Activating *MAPK1* (ERK2) mutation in an aggressive case of disseminated juvenile xanthogranuloma

SUPPLEMENTAL DATA

Karyotype results

Summary:

1) Abnormal chromosome analysis showing complex chromosomal rearrangements.

2) Abnormal FISH analysis showing three copies of the *IGH* gene.

Chromosome analysis of left cervical lymph node revealed clonal aberrations in 8 out of 21 cells examined. A stemline showing an apparently reciprocal 3-way translocation between chromosome 2 homologues at bands 2p15 and 2q33 and chromosome 15q24, additional material on chromosomes 5q35, 18q23 and 22p11.1, an apparently reciprocal translocation between the chromosome 5 homologue at band 5q13 and 6q23, as well as chromosomes 14q11.2 and 19q13.3, deletion of chromosome 11p13-p15, loss of chromosome 13, and gain of a probable inversion of chromosome 19 at bands 19p13.3-q12, was observed in all abnormal cells. A sideline showing deletion of chromosome 5 at bands 5q22-q35 (in addition to the two abnormal copies of 5 mentioned in the stemline clone), was observed in four cells. Metaphase fluorescence *in situ* hybridization (FISH) using the *IGH* break apart probe at 14q32 and telomere probes specific for chromosomes 2, 5, 6, 13 and 19 on previously G-banded cells confirmed the t(2;2;15), add(5q), t(5;6), -13 and the t(14;19), as described above. FISH analysis revealed the probable inv(19) as a derivative chromosome 19 resulted from an inversion and insertion of *IGH* gene (14q32) into the short arm of chromosome19. The remaining thirteen cells showed a normal male karyotype.

Concurrent fluorescence *in situ* hybridization (FISH) evaluation using a dual color, break apart probe set specific for *IGH* gene revealed three copies of the *IGH* gene, in 95 out of 200 (47.5%) interphase cells examined. In addition, FISH evaluation using a dual color probe set specific for chromosome 9 centromere and *CDKN2A* (p16) gene region, as well as a dual color, break apart probe set specific for the *TCF3* (E2A) gene at 19p13 revealed no of evidence *CDKN2A* gene deletion or *TCF3* gene rearrangement in 400 total interphase cells examined. All controls were appropriate.

The FISH tests performed were specific for detecting *CDKN2A* (p16) gene deletion, *IGH* and *TCF3* gene rearrangements, as well as copy numbers, of all loci tested. Other unrelated chromosomal anomalies will not be detected using these tests.

SUPPLEMENTARY FIGURE AND TABLES

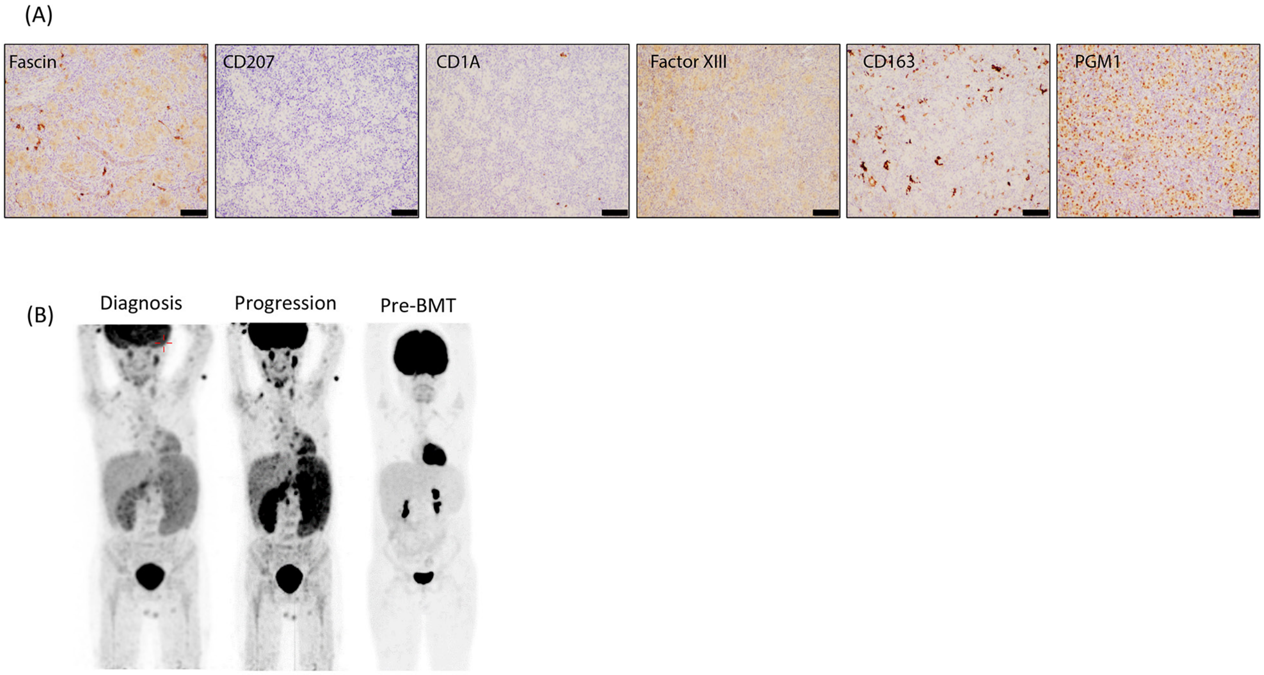

Supplementary Figure 1: (A) Immunohistochemistry of a post-chemotherapy lymph node biopsy with horseradish peroxidase-conjugated secondary antibody was used to identify expression of markers typical for dendrocytes and macrophages**.** Images were obtained using the Olympus BX51 microscope. Scale bar represents 100 µm. **(B)** Representative 18-FDG PET images at diagnosis, progression, and posttreatment with dexamethasone/etoposide prior to hematopoietic stem cell transplant, when the patient had persistent hepatosplenomegaly but no PET-avid disease.

Supplementary Table 1: Summary of clinical history and management of the patient

See Supplementary File 1

Supplementary Table 2: Somatic mutations identified in the JXG patient by whole exome sequencing

Gene	Chromosome Location	cDNA Change	AA Change	Exome Sequencing			
				Tumor Total Coverage	Tumor Variant Coverage	Normal Total Coverage	Normal Variant Coverage
MAPK1	chr22:22127167	c.G961A	p.D321N	71	7	102	0
CD3E	chr11:118183345	c.C116G	p.S39C	145	11	220	0
FRY	chr13:32863874	c.G8574T	p.M2858I	81	4	113	0
ZNF676	chr19:22363737	c.G782C	p.G261A	32	5	42	0

cDNA, complementary DNA; *AA*, amino acid.
