# Supplementary material for: Activating MAPK1 (ERK2) mutation in an aggressive case of disseminated juvenile xanthogranuloma
Source: Oncotarget. 2017 Apr 29;8(28):46065–70. doi: 10.18632/oncotarget.17521 (PMC5542249; doi:10.18632/oncotarget.17521)
Supplement: Supplementary file 2 [file oncotarget-08-46065-s002.docx]

**SUPPLEMENTAL TABLES**

**Supplemental Table 1. Summary of clinical history and management of the patient.**

| Date | Clinical findings and treatment | Radiology | Pathology | Research Analysis |
| --- | --- | --- | --- | --- |
| August 2012 | Initial presentation |  | **LYMPH NODE:** epithelioid granulomas (high lysozyme, paranuclear CD68, low CD163) |  |
| June 2013 | Clinical course: Fever, lymphadenopathy, hepatosplenomegaly, cytopenias  Rx: Clofarabine x two cycles  Outcome: PR (significant decrease in splenomegaly and adenopathy after one cycle) | **PET/CT:**  FDG avid cervical and supraclavicular lymphadenopathy, portal hepatis lymphadenopathy. Splenomegaly with diffuse, heterogeneous, increased FDG uptake. | **LYMPH NODE:** epithelioid histiocytic infiltrate, poorly formed granulomas (high lysozyme, paranuclear CD68, low CD163).  **Flow cytometry:** T cell population shows reversal of CD4:CD8 ratio and partial loss of CD7.  **FISH:** Trisomy IgH copy number change (47.5%) of interphase cells.  **BONE MARROW:**  Focal patchy areas of histiocytic proliferation mixed with small lymphocytes. Normocellular with trilineage hematopoiesis.  **Flow cytometry:** T cells show partial loss of CD7  **FISH:** No abnormal IgH findings |  |
| August 2013 | Clinical course: Interval progression of lymph node disease  Rx: Therapy changed to ALCL-99 x2 courses with methotrexate, etoposide, ifosfamide, dexamethasone  Outcome: PR, decrease number/size of cervical and occipital lymph nodes, decreased spleen size | **PET/CT:**  Interval improvement in FDG activity in spleen but overall progression of disease with increased and new FDG activity in multiple regions of lymph nodes bilaterally and on both sides of the diaphragm (cervical, axillary, mediastinal, porta hepatis, retroperitoneum, mesenteric, iliac, inguinal) | **LYMPH NODE:**  **FISH:** Trisomy IgH copy number change (61%) of interphase cells.  **BONE MARROW:**  Patchy histiocytic proliferation with formation of non-caseating granulomata. Decreased trilineage hematopoiesis.  Inguinal lymph node: Extensive histiocytic proliferation with formation of non-caseating granulomata.  **Flow cytometry:**  T cell population showing partial loss of CD7 and virtual absence of mature B cells.  **FISH:** Trisomy IgH copy number change (6%) of interphase cells. | **RESEARCH LESION ANALYSIS (Aug 29, 2013)** |
| Sept 2013 | Clinical course: Fluctuating adenopathy, splenomegaly, cytopenias |  | **BONE MARROW:**  Histiocytic proliferation and hypocellular marrow with decreased trilineage hematopoiesis.  **Flow cytometry:**  T cell subset with loss of CD7, increased NK cells, increased monocytes, virtual absence of B cells.  **FISH:** Trisomy IgH copy number change (5.5%) of interphase cells. |  |
| October 2013 | Outcome: PD in cervical and chest lymph nodes, stable abdominal lymph nodes, spleen and liver  Rx: alemtuzumab x 5 days | **PET/CT:**  Disease progression with interval increase in size and activity of multiple lymph nodes throughout the body. Focal uptake in the right renal parenchyma. Stable splenic involvement of disease. |  |  |
| November 2013 | Outcome: PR, no adenopathy, persistent hepatosplenomegaly  Rx: alemtuzumab x one dose |  |  |  |
| December 2013 | Outcome: PD, worsening hepatosplenomegaly, cytopenias, pleural effusion requiring chest tube  Rx: Therapy changed to bortezomib, ifosfamide, vinorelbine (received two cycles); developed significant renal toxicity with Fanconi syndrome | **PET/CT:** Mixed treatment response with overall marked improvement in the FDG avidity within multiple lymph nodes throughout the body. Increased activity within the enlarged liver and spleen suggestive of focal disease superimposed on diffuse disease. | **PLEURAL FLUID:**  Abundant RBCs with occasional lymphocytes, monocytes, and mesothelial cells.  **Flow cytometry:** T cell subset with loss of CD7, increased NK cells, absent B cells. |  |
| January 2014 | Clinical course: PD, splenomegaly worsening  Rx: Start HLH-1994 therapy with etoposide and dexamethasone x three months  Outcome: PR, significant improvement in hepatosplenomegaly, cytopenias slightly better |  | **BONE MARROW:** No histiocytic proliferation. Markedly hypocellular marrow with decreased trilineage hematopoiesis.  **Flow cytometry:**  T cell subset with loss of CD7, increased NK cells, increased monocytes, virtual absence of B cells.  **FISH:** IgH copy number change (4%) of interphase cells. |  |
| Early April 2014 | Clinical course: PR, significant improvement in hepatosplenomegaly, cytopenias slightly better | **PET/CT:** Hepatosplenomegaly without abnormal FDG uptake. No evidence of abnormal hypermetabolic foci throughout the neck, chest, abdomen, and pelvis. | **BONE MARROW (early April 2014):** No abnormal histiocytic aggregates. Hypocellular marrow with trilineage hematopoiesis. **Flow cytometry:**  Absent B and T cells.  **FISH:** No abnormal IgH copy number. |  |
| Late April 2014 | Relapse, recurrent fevers, worsening splenomegaly  Rx: dexamethasone and etoposide restarted |  | **BONE MARROW (late April 2014):** No abnormal histiocytic aggregates. Variably cellular bone marrow with erythroid predominant maturing trilineage hematopoiesis.  **FISH:** Trisomy IgH copy number change (2%) of interphase cells |  |
| June 2014 | Splenomegaly improving  Rx: HSCT with MMUD and conditioning regimen of busulfan, cyclophosphamide, fludarabine, alemtuzumab |  | **BONE MARROW:** No abnormal histiocytic aggregates identified. Markedly hypocellular marrow with trilineage hematopoiesis and erythroid predominance.  **FISH:** Trisomy IgH copy number change (2.5%) of interphase cells. |  |
| July 2014 |  |  | **BONE MARROW:** No abnormal histiocytic aggregates identified. Markedly hypocellular, damaged marrow (less than 5%) with panhypoplasia and left shifted myelopoiesis.  **FISH:** No abnormal IgH findings |  |
| August 2014 | Engrafted with 100% donor cells. Bone marrow positive for parvovirus and HHV6.  Patient developed respiratory failure thought to be secondary to donor immune response vs infection, fluid overload, and ultimately shock with refractory hypotension leading to death. |  | **BONE MARROW:** No abnormal histiocytic aggregates identified. Markedly hypocellular, damaged marrow (less than 5%) with panhypoplasia and left shifted myelopoiesis.  **FISH:** No abnormal IgH findings |  |
